# Ultrasonographic Findings of Polyacrylamide Gel as a Filler in the Gluteal Region

**DOI:** 10.1111/jocd.70518

**Published:** 2025-10-20

**Authors:** Eliza Porciuncula Justo Ducati, Fernanda Aquino Cavallieri, Gabriel de Sousa Lima, Camila Souza de Araujo Guarinello, Ana Letícia Barbosa do Rego, Claudia Regina dos Santos Fortes

**Affiliations:** ^1^ ED Ultrassonografia Dermatológica Sao Paulo Brazil; ^2^ Cavallieri Diagnóstico Por Imagem Rio de Janeiro Brazil; ^3^ Blanc Hospital Sao Paulo Brazil

To the Editor,

The use of injectable fillers has grown significantly in recent decades, with procedures increasing from 1.6 million in 2011 to over 2.4 million in 2015 [1]. Various products with different biochemical properties have been employed, influencing tissue response [2]. Among these, polyacrylamide gel (PAAG) stood out from the 1990s to the mid‐2010s due to its long‐lasting volumizing effect and perceived biocompatibility. However, its late‐onset complications have become increasingly evident.

The rise in the use of permanent soft‐tissue fillers such as PAAG for gluteal augmentation has been met with growing concerns over late‐onset complications, including asymmetry, migration, granuloma formation, and infection [1, 3, 4]. Ultrasonography (US) offers a rapid, non‐invasive method to identify and characterize these adverse events, yet reports on its application in the gluteal region remain limited [2, 5]. We herein present three novel US patterns of PAAG in 10 patients and discuss their implications for clinical management.

Between June 2023 and October 2024, 10 women (mean age 38 years, range 29–52) with a history of gluteal PAAG injections underwent high‐frequency US (4–22 MHz) mapping of the cutaneous, subcutaneous, and muscular layers. Images were independently reviewed by a radiologist. Representative cases were selected to illustrate characteristic findings. The examinations were performed using a GE Logic machine with multifrequency probes, starting with a 6–15 MHz linear probe in the upper quadrants (lateral and medial consecutively), followed by the junction of the quadrants and then the medial and lateral quadrants. There was only one radiologist. On average, there were 8 years between the injection and the examination.

Three distinct US patterns were identified. Overall, cases presenting collections on ultrasound demonstrated a higher propensity for migration and fistulization toward the dermis, and patients with this pattern were more frequently considered eligible for surgical product removal. Nevertheless, all cases still exhibited residual material in post‐procedure examinations, reinforcing the notion that PAAG behaves as a permanent filler often associated with complications. The sonographic appearances included elongated “glove‐finger” collections, characterized by hypoechoic, slightly heterogeneous tracks with smooth margins following fibrous septa (Figure 1); round hypoechoic–isoechoic nodules with hyperechoic deposits, appearing as well‐defined spherical structures containing punctate echogenic foci, likely reflecting internal gel fragmentation or admixture with other fillers (Figure 2A); and conglomerated pseudocystic areas, consisting of clustered hypoechoic cavities with internal hyperechoic flecks, occasionally adjacent to anechoic vesicles, consistent with chronic inflammatory remodeling and loculation (Figure 2B).

**FIGURE 1:**
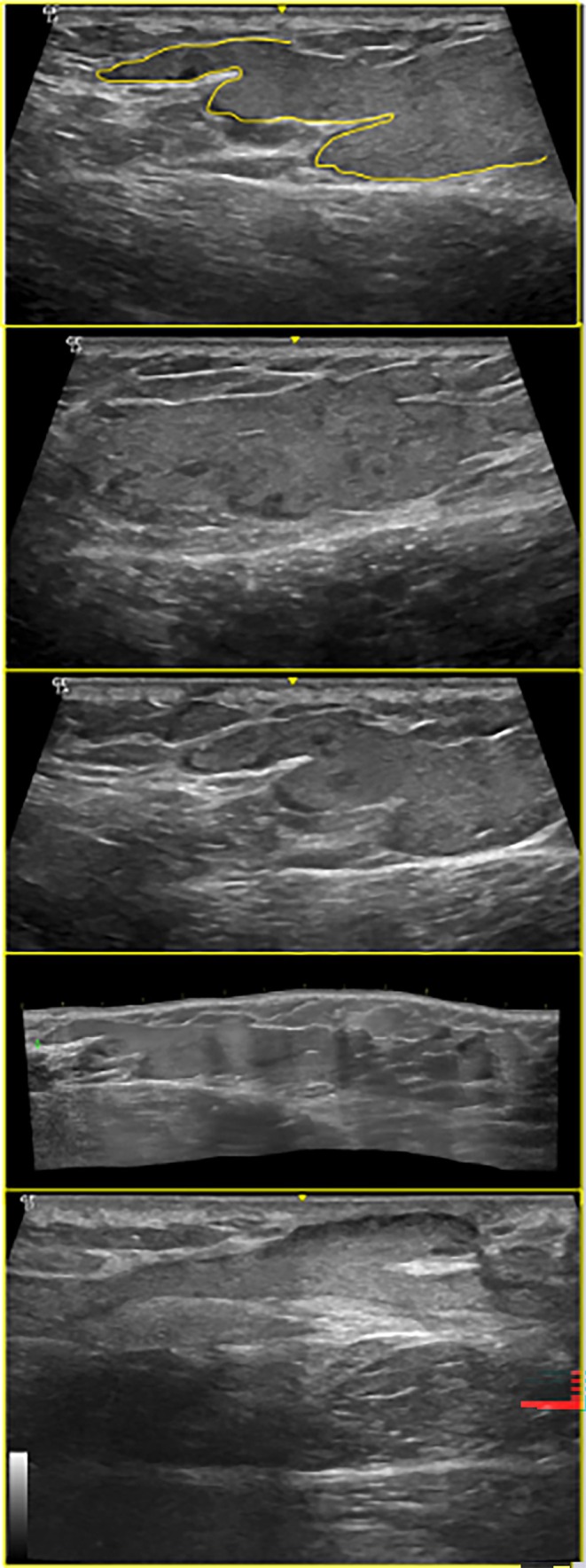
Linear, hypoechoic “glove‐finger” tracks of PAAG along fibrous septa (6–15 MHz linear probe).

**FIGURE 2:**
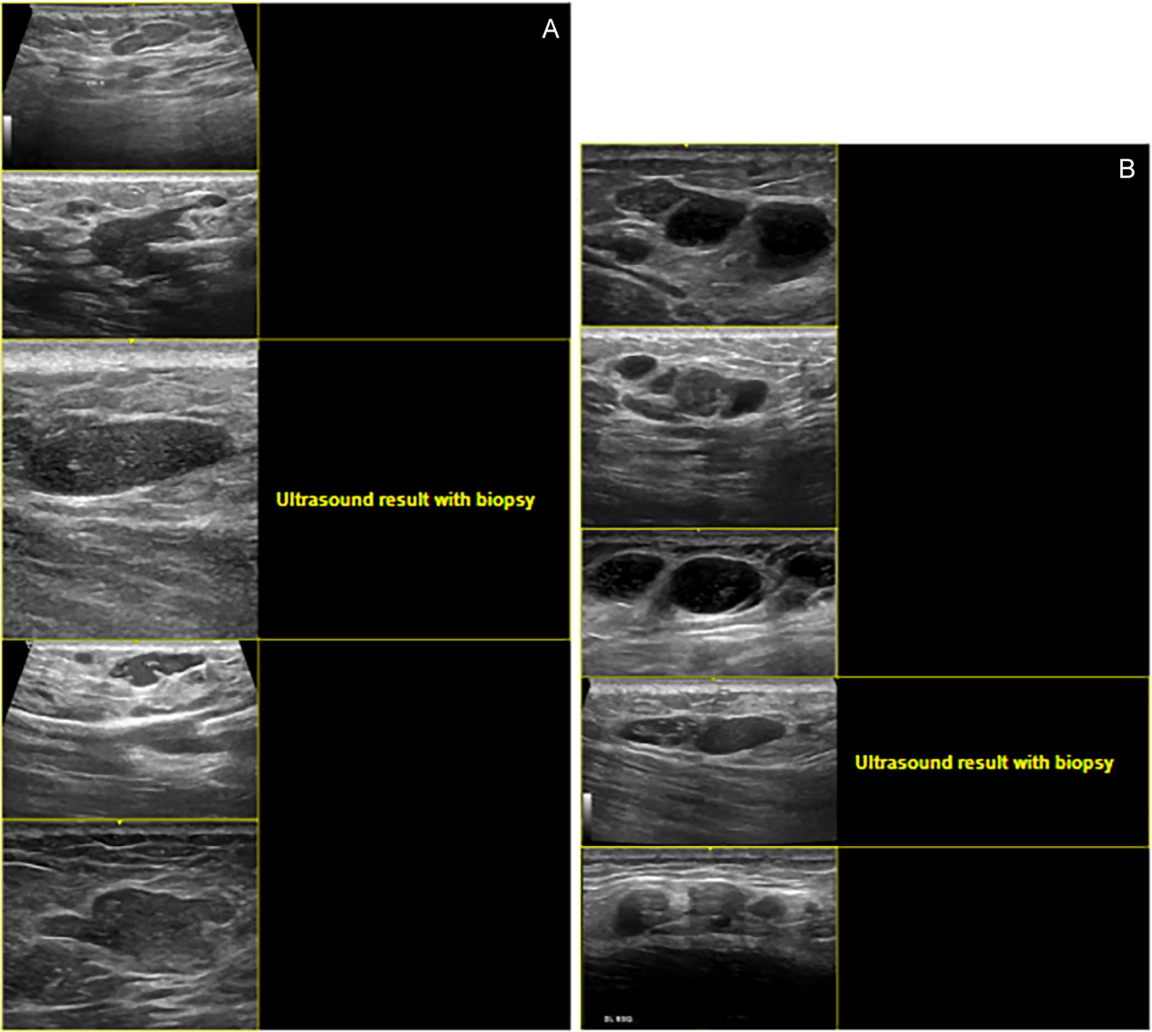
(A) Round hypoechoic–isoechoic nodules with internal hyperechoic deposits. (B) Conglomerated pseudocystic areas with hyperechoic flecks.

Histopathology in two cases confirmed amorphous material with Alcian blue positivity and calcium deposits on von Kossa staining, corroborating US findings and indicating long‐standing PAAG presence.

These patterns expand upon previously reported cystic and pseudocystic appearances of PAAG [6], demonstrating that gel distribution may present as linear infiltrations, discrete nodules, or complex loculated masses. Recognizing these sonographic signatures is critical for differentiating PAAG from other fillers (e.g., hyaluronic acid) and guiding interventions such as ultrasound‐guided aspiration, surgical excision, or medical therapy for inflammatory reactions. Among the many injectables previously approved and those still currently used for body fillers is hyaluronic acid, which up to now has been described as the main differential diagnosis of PAAG, since both can appear on ultrasound as rounded hypoechoic or anechoic areas. However, hyaluronic acid changes in size and shape over time, whereas PAAG does not. Silicone and polymethylmethacrylate (PMMA) are two other permanent substances frequently found in gluteal mapping exams. On ultrasound, silicone appears as a hyperechoic substance, usually located in the subdermal layer, often presenting with a “snowstorm” artifact and sometimes interspersed with anechoic vesicles representing pure silicone. PMMA, on the other hand, presents as an irregular, hyperechoic, conglomerated substance with posterior acoustic shadowing artifact (when located in the subcutaneous tissue).

US is a valuable diagnostic and monitoring tool for PAAG complications in the gluteal region. Familiarity with the “glove‐finger” tracks, nodular formations with hyperechoic deposits, and conglomerated pseudocystic areas enhances detection accuracy and informs tailored treatment strategies. High‐frequency US is the gold standard method for identifying and differentiating injectable substances. Professionals who are skilled in performing this type of examination must have detailed knowledge of the characteristics of each product, which significantly increases the specificity of the assessment.

## Author Contributions

**Eliza Porciuncula Justo Ducati:** contributed to the search for scientific articles, the writing of this article, the review of the content and the suggestion of revisions, the conduct and analysis of imaging examinations, and the description of ultrasonographic findings. **Fernanda Aquino Cavallieri:** contributed to the search for scientific articles and the writing of this article. **Claudia Regina dos Santos Fortes:** contributed to the search for scientific articles and the writing of this article. **Ana Letícia Barbosa do Rego:** contributed to the search for scientific articles and the writing of this article. **Gabriel de Sousa Lima:** contributed to the search for scientific articles and the writing of this article. **Camila Souza de Araujo Guarinello:** contributed to the search for scientific articles and the writing of this article.

## Ethics Statement

We also emphasize that all stages of the research were conducted independently, ethically, and transparently, with a full commitment to scientific integrity, the protection of the rights and safety of the participants, in strict compliance with CNS Resolution No. 466/12 and other applicable ethical guidelines for research involving human beings. This study involved human subjects, but does not include a statement of informed consent.

## Conflicts of Interest

The authors declare no conflicts of interest.

## Data Availability

Research data are not shared.
